# Effect of GnRH Active Immunisation on Reproductive Performance of Male Sprague Dawley Rats

**DOI:** 10.3390/ijms25063193

**Published:** 2024-03-11

**Authors:** Cheng-Qi Zhang, Shuang-Shuang Li, Bo Hu, Li-Wen Xu, Jia-Jia Liu, Ya-Jie Sun, Xue Bai

**Affiliations:** Key Laboratory of Special Animal Epidemic Disease, Ministry of Agriculture, Institute of Special Animal and Plant Sciences, Chinese Academy of Agricultural Sciences, Changchun 130112, China; zcq971102@163.com (C.-Q.Z.); isliujiayuan@126.com (J.-J.L.);

**Keywords:** GnRH, active immunisation, testosterone, rats

## Abstract

To investigate the effect of active immunisation with gonadotropin-releasing hormone (GnRH) on the reproductive function in male Sprague Dawley (SD) rats, 24 42-day-old rats were randomly assigned to treatment with GnRH6-MAP, GnRH-OVA, a surgical castration group, and a blank control group. Each rat in the treatment groups was intramuscularly injected at 6, 8, and 10 weeks of age. The serum concentrations of testosterone (T), follicle-stimulating hormone (FSH), luteinising hormone (LH), and anti-GnRH antibodies were determined using enzyme-linked immunosorbent assays. The results showed that active immunisation with recombinant GnRH6-MBP and GnRH-OVA significantly increased the serum levels of anti-GnRH antibodies and reduced the serum concentrations of testosterone compared to the black control. Eight weeks after immunisation, the rats’ testes were surgically removed for morphological evaluation, showing atrophy of the convoluted vasculature, relative emptying of the lumen, and insignificant differentiation of spermatogonial cells, which were increased in weight and volume compared with the blank control group. These findings indicated that active immunisation with GnRH can lead to testicular atrophy and reduce gonadal hormone concentrations, suggesting that GnRH is a highly effective immunogen.

## 1. Introduction

Immunocastration is a paradigm shift in animal birth control strategies. This approach relies on vaccination against the gonadotropin-releasing hormone (GnRH) to inhibit reproductive functions [1,2]. GnRH, a pivotal neuropeptide synthesised and secreted by the hypothalamus [3], plays a central role in regulating the reproductive axis. Structurally, GnRH is a decapeptide with a specific sequence that is pyroglutamylated and amidated at its terminus, conferring enhanced biological activity and stability [1]. The synthesis and pulsatile release of GnRH by hypothalamic neurones and its subsequent transport to the pituitary gland through the portal system underscore its significance in the endocrine orchestration of reproduction [4,5]. The hypothalamic arcuate nucleus, influenced by a plethora of factors such as sex steroids and neurotransmitters, houses a GnRH pulse generator that is fundamental for the modulation of this system [6].

Because of the inherently weak immunogenicity of GnRH monomers, they have been conjugated with various carrier molecules such as ovalbumin (OVA), keyhole limpet haemocyanin (KLH), and bovine serum albumin (BSA) to bolster vaccine efficacy [7]. Previous studies have demonstrated that active immunisation with a maltose-binding protein-GnRH6 fusion (GnRH6-MBP) markedly reduced the reproductive potential of mice and boars, highlighting the effectiveness of this vaccine [8,9,10].

Upon binding to its receptors on pituitary gonadotrophs, GnRH induces the secretion of luteinising hormone (LH) and follicle-stimulating hormone (FSH), which are crucial for reproductive functions [11]. This interaction triggers a cascade of intracellular events via the G protein-coupled phospholipase C pathway, culminating in the release of intracellular calcium ions and the secretion of LH and FSH [12,13]. Such hormonal stimulation is essential for the development of gonads and the synthesis of testosterone (T), as demonstrated in previous studies [14]. Conversely, Han et al. showed that GnRH tandem-dimer peptide immunisation reduced the levels of T, LH, and FSH in male rats and rams, indicating the suppression of the reproductive axis [15,16].

In addition to its endocrine effects, GnRH influences the morphological aspects of animal growth and development. Jiang et al. observed seminal tubular hypoplasia and reduced spermatogonial differentiation in immunised mice, confirming the profound morphological impact of GnRH-targeted immunisation [3]. Similarly, chitosan-adjuvant GnRH vaccines are associated with testicular atrophy and the onset of azoospermia, suggesting their effectiveness in inducing sterility [17]. Liu et al. observed that immunisation with GnRH6-MBP led to testicular shrinkage and degeneration in canines [18].

The indirect enzyme-linked immunosorbent assay (ELISA) is a widely employed technique for the detection of antibodies and is renowned for its rapidity, high sensitivity, exceptional specificity, and straightforward methodology [19]. Based on these attributes, we developed an indirect ELISA protocol for quantifying GnRH-MBP antibodies in rat serum. Our decision to utilise this approach was geared towards facilitating the detection of GnRH6-MBP antibodies, thereby laying the groundwork for future rat immunisations with recombinant GnRH6-MBP proteins. The establishment of this assay is instrumental in providing the necessary conditions for immunological evaluation in rats and ensuring a solid foundation for the advancement of vaccine research and development.

This study aimed to elucidate the efficacy of two recombinant GnRH fusion proteins in curtailing testicular development and reducing T, LH, and FSH levels. The specific principle is that by immunising rats with exogenous GnRH, the body induces the production of anti-GnRH antibodies, which neutralise endogenous GnRH, resulting in their castration. Here, we aimed to shed light on the immunological mechanisms underlying GnRH-based immunosterilisation, thereby contributing to a comprehensive and systematic understanding of animal sterilisation. Our results advance the practical deployment of GnRH immunosterilisation vaccines, fostering a humane and effective approach to animal population control.

## 2. Results

### 2.1. Identification of Recombinant Plasmid

**Figure 1:**
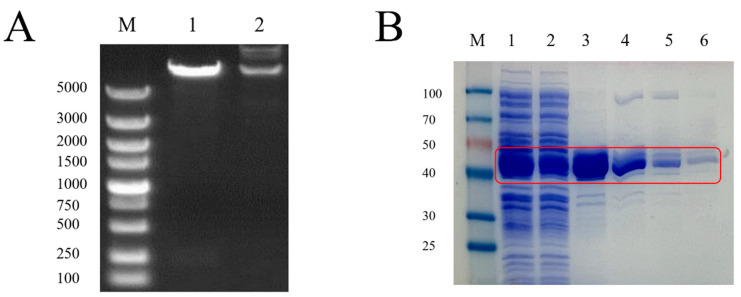
(**A**) Identification of recombinant plasmids by enzyme digestion. Lane M: DNA Marker (DL5000); Lane 1: Plasmid digested by *EcoR*I *SaI*I; Lane 2: Plasmid DNA (GnRH). (**B**) GnRH protein purification map. Red box: GnRH6-MBP protein. M: Protein Marker; 1: pMAL-c2x-GnRH supernatant induced by IPTG; 2: GnRH6-MBP solution; 3: GnRH6-MBP eluent 1; 4: GnRH6-MBP eluent 2; 5: GnRH6-MBP eluent 3; 6: GnRH6-MBP eluent 4.

The products of double-enzyme digestion of the recombinant plasmid with *EcoR*I and *Sal*I and the recovered products of double-enzyme digestion of the plasmid pMAL-c2x and GnRH6 gene were electrophoresed simultaneously on an agarose gel. The results showed that the lengths of the two enzyme-digested fragments of the recombinant plasmid were equal to those of the enzyme-digested fragments of the plasmid pMAL-c2x and the GnRH6 gene, respectively (Figure 1A).

### 2.2. Identification of Recombinant Protein

SDS-PAGE analysis showed that the prokaryotically expressed pMAL-c2x-GnRH6 recombinant protein mainly existed in the supernatant form, with a molecular weight of approximately 45 kDa, and it exhibited high purity after purification with Dextrin Beads 6FF (Figure 1B).

### 2.3. Identification of Synthetic Protein

**Figure 2:**
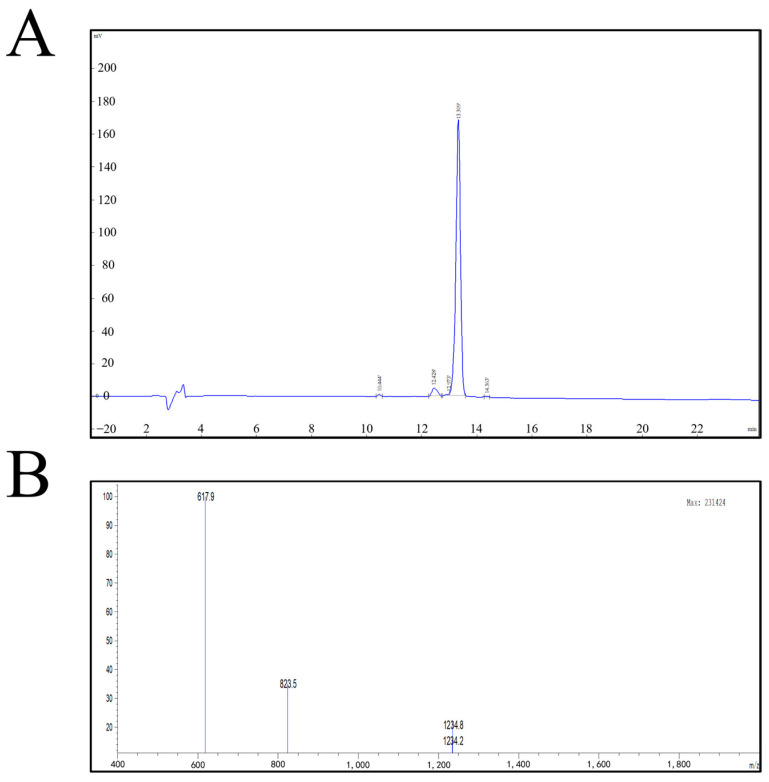
(**A**) GnRH synthetic peptide HPLC chromatographic column results. (**B**) GnRH synthetic peptide mass spectrometry report.

The purity of the synthetic peptide was measured by HPLC, and the purity was >98% (Figure 2A). The molecular weight was determined to be 2467.72 Da according to mass spectrometry (MS) (Figure 2B).

### 2.4. Establishment of an ELISA Method for GnRH6-MBP Antibody Detection

The chequerboard titration method was used to determine the ideal serum dilution and antigen coating concentration for the assay. According to Table 1, the highest positive-to-negative absorbance ratio (P/N) at a wavelength of 450 nm was achieved with an antigen coating concentration of 2 μg/mL (100 μL/well) and a serum dilution of 1:10, yielding a P/N value of 10.896. Consequently, the optimal GnRH-MBP protein coating concentration was established at 2 μg/mL, and the serum was optimally diluted at 1:10. Furthermore, Table 2 indicates that a P/N ratio of 12.297, the peak for the assay, was obtained when the enzyme-conjugated antibody was diluted to 1:10,000. Therefore, this dilution was selected as optimal for the enzyme-conjugated antibody. Data in Table 3 reveal that using a 50 g/L skim milk powder solution as the blocking agent resulted in the highest P/N ratio of 12.822 at the wavelength of 450 nm, designating it as the preferred blocking solution. As depicted in Table 4, the antigen coating condition of 4 °C for 12 h yielded a superior P/N ratio of 13.067, establishing these parameters as the most favourable antigen coating conditions. Finally, Table 5 demonstrates that a TMB colour development duration of 20 min led to the highest P/N ratio of 16.050. Therefore, the optimal colour development time for TMB was determined to be 20 min.

### 2.5. Results of ELIS, and Sensitivity and Repeatability Tests

After testing 10 copies of GnRH6-MBP-negative serum samples, the average OD450 nm was found to be 0.121 with a standard deviation of 0.011. According to the formula for calculating critical values (X̄ + 3SD), the critical value for distinguishing between negative and positive samples was 0.154. Consequently, test samples with OD450 nm > 0.154 were considered positive, those with OD450 nm < 0.154 were considered negative, and samples with OD450 nm = 0.154 were considered indeterminate.

The detection of 10 GnRH-MBP positive serum samples and the positive and negative serum samples was undertaken at a wavelength of 450 nm, and the absorbance value ratio (P/N) was greater than 0.154. The average OD value was 0.229 at a dilution ratio of 1:81,920.

As shown in Table 6, the lowest coefficient of variation of the intra-batch repeated tests was 1.614%, and the highest was 4.212%. The coefficients of variation for the repeated tests between batches were 0.694% and 5.358%, both of which were lower than 10.0%. The results show that the proposed method exhibits good repeatability.

### 2.6. Antibody Detection in Rat Serum

**Figure 3:**
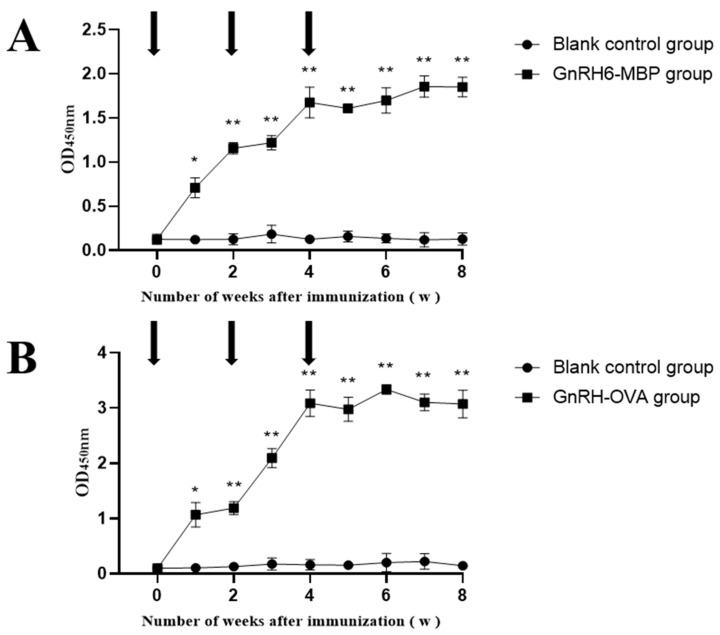
Changes of antibody in serum. The arrow indicates the time point of each immunisation; * = *p* < 0.05 significant difference; ** = *p* < 0.01 significant difference. (**A**): GnRH-MBP antibody levels in rat serum; (**B**): GnRH-OVA antibody levels in rat serum.

Two weeks after the first immunisation, the antibody titres of the GnRH6-MBP group and GnRH-OVA group were significantly higher than those of the blank control group (*p* < 0.05) and continued until 4 weeks after the third immunisation (Figure 3).

### 2.7. Changes in the Weight of Rat Testes and Observations of Tissue Sections

**Figure 4:**
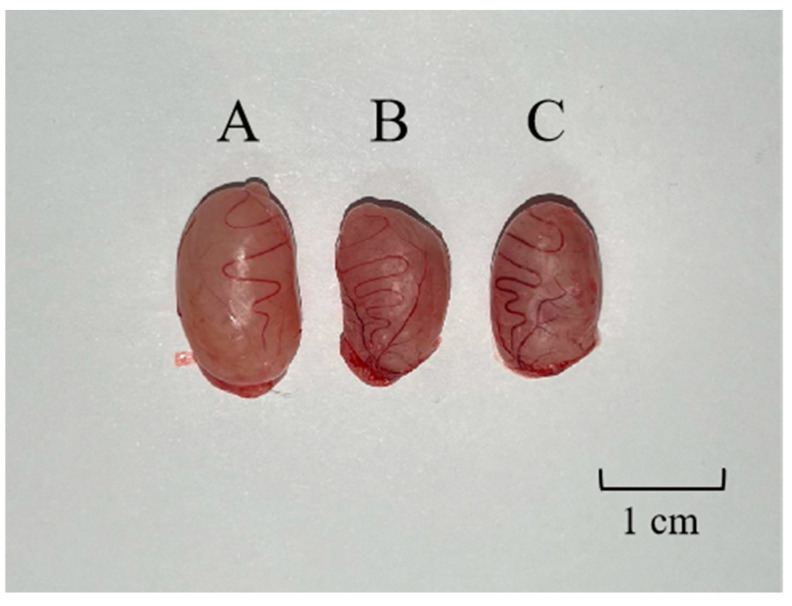
(**A**) Testicular contrast map. One testis was randomly selected in the blank control group, the GnRH-MBP group, and the GnRH-OVA group, respectively. (**A**) Testes of blank control group rat, 1.496 g; (**B**) testes of the GnRH-MBP group rat, 1.032 g; (**C**) testes of the GnRH-OVA group, 1.101 g.

The weights and volumes of rat testes in the GnRH6-MBP and GnRH-OVA groups were significantly lower than those in the blank control group (Figure 4). The weights and volumes of rat testes in the GnRH-OVA group were slightly lower than those in the GnRH6-MBP group.

**Figure 5:**
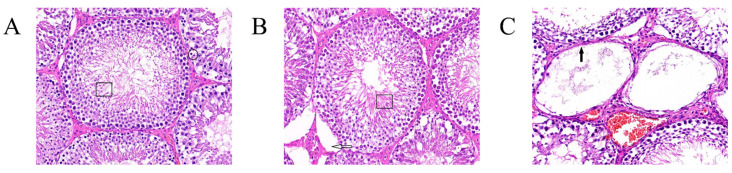
Testicular tissue sections and H&E staining (400×). (**A**) Blank control group, box: regular arrangement of spermatogenic cells at all levels in the convoluted testicular spermatid tubule., circle: a high number of spermatogonia; (**B**) GnRH-OVA group, box: regularly arranged spermatogenic cells in testicular seminiferous tubules at all levels, hollow arrows: interstitium was slightly sparse; (**C**) GnRH-MBP group, solid arrow: no spermatozoa and few spermatogonia occurred in the seminiferous tubules.

**Figure 6:**
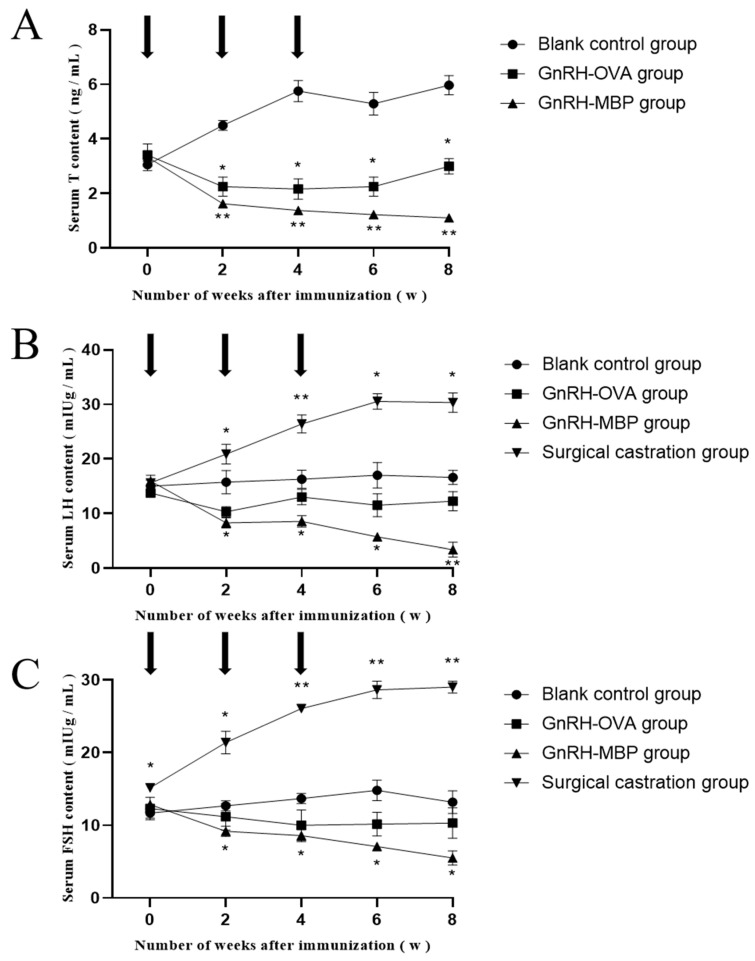
The changes of T, LH, and FSH in serum. (**A**) determination of T content in serum; (**B**) determination of LH content in serum; (**C**) determination of FSH in serum. The arrow indicates the time point of each immunisation; * = *p* < 0.05 significant difference; ** = *p* < 0.01 significant difference.

Spermatogenic cells at all levels of the convoluted testicular spermatid tubules in the blank control group were regularly arranged (Figure 5A, box), filled the lumen, and had a high number of spermatogonia (circle). Spermatogenic cells at all levels of the testicular seminiferous tubules in the GnRH-OVA group were regularly arranged (Figure 5B, boxes), and the interstitium was slightly sparse (hollow arrows). No spermatozoa and few spermatogonia occurred in the seminiferous tubules of the GnRH6-MBP group (Figure 6C). The spermatogonia at all levels were detached and disappeared due to apoptosis, and only supporting cells and spermatogonia against the basement membrane were seen in some of the seminiferous tubules (solid arrows).

### 2.8. Changes in Serum T, LH, and FSH

Serum T concentrations in the GnRH6-MBP and GnRH-OVA groups were significantly lower than those in the blank control group at 2 weeks after the first immunisation (*p* < 0.05; Figure 6A) and remained as such until 8 weeks after the first immunisation. There was almost no difference between the GnRH6-MBP and GnRH-OVA groups.

Serum LH concentrations in the GnRH6-MBP and GnRH-OVA groups were significantly lower than those in the blank control group at 2 weeks after the first immunisation (*p* < 0.05; Figure 6B) and remained as such until 8 weeks after the first immunisation. There was almost no difference between the GnRH6-MBP and GnRH-OVA groups.

Serum FSH concentrations in the GnRH6-MBP and GnRH-OVA groups were significantly lower than those in the blank control group 2 weeks after the first immunisation (*p* < 0.05; Figure 6C). Eight weeks after the first immunisation, almost no differences were observed between the GnRH6-MBP and GnRH-OVA groups.

## 3. Discussion

Human immune castration can avoid the physical harm caused by surgery and interfere with the disease caused by sex hormone levels [20]. In hormone-sensitive diseases such as advanced prostate cancer, castration significantly hinders disease progression by lowering testosterone levels, thereby prolonging patient survival and relieving symptoms associated with tumour growth, such as pain and dysuria [21,22]. In gender-affirming therapy for transgender individuals, castration forms an integral part of the transition process that helps suppress endogenous sex hormone levels and aligns physical characteristics more closely with the individual’s gender identity [23,24].

Urbanisation and modernisation of society have led to an increase in pet ownership, necessitating effective animal population management strategies [25]. Castration is widely implemented to reduce the number of stray animals, reduce urban management challenges, and enhance animal welfare; however, traditional surgical castration has the disadvantages of surgical risks, anaesthetic side effects, postoperative pain, and extended recovery periods, limiting its broader application [26]. Castration, by removing an animal’s reproductive organs, not only controls population growth but also curtails aggressive behaviours, particularly in males while females are in oestrus. This aggression poses risks to human safety and animal health, as animals may harm themselves or others. Moreover, castration can modify temperament and behaviour, which better corresponds with human requirements for companion animals, such as reduced territorial markings and increased docility [27].

Recombinant protein production is a cornerstone in biotechnology, with eukaryotic and prokaryotic systems being employed based on the nature of the required protein. Prokaryotic expression systems, especially in *E. coli*, are favoured because of their simplicity, cost-effectiveness, and rapid production capabilities [28]. This study utilised an *E. coli* system to express the recombinant GnRH6 protein, leveraging the pMAL-c2x vector for its multiple cloning sites and the inclusion of an MBP tag to enhance solubility and folding efficiency in prokaryotic cells [29].

The recombinant GnRH6 protein, with its relatively short sequence, was successfully expressed as a fusion protein with an MBP tag, facilitating correct folding and solubility. The induction of this protein under IPTG and the strong ‘tac’ promoter resulted in high-level expression. Subsequent affinity purification using dextrin MBP protein chromatography medium (6FF) yielded a high-purity protein, as evidenced by SDS-PAGE and HPLC, with the molecular weight confirmed via MS.

Furthermore, the use of the one-step fluorescence qPCR kit in this study demonstrates the importance of meticulous reagent preparation and handling to ensure accuracy and minimise contamination. This precision was crucial for assessing the effectiveness of GnRH proteins in inducing immunological castration in male SD rats.

Histological examination through haematoxylin/eosin staining confirmed the absence of sperm in the seminiferous tubules of the treated groups, indicating the effectiveness of GnRH6-MBP and GnRH-OVA in inducing sterility. Immunisation led to testicular atrophy, a significant decrease in mRNA levels of LH receptor and FSH receptor, and the absence of sperm cell apoptosis, confirming the impact of GnRH proteins on the reproductive physiology of rats.

This study contributes to a broader understanding of the role of animal castration in urban animal management and the potential of recombinant protein technology for developing non-surgical sterilisation methods. While promising, these findings also highlight the need for further studies to refine these techniques and assess their long-term effects on animal health and behaviour.

The pMAL-c2x-GnRH6 recombinant plasmid was successfully prepared using agarose gel electrophoresis. The GnRH6-MBP recombinant protein was successfully expressed in a prokaryotic system. After purification, purity was verified by SDS-PAGE. A GnRH synthetic peptide was successfully obtained, and purity exceeded 98% using HPLC. The molecular weight was determined to be 2467.72 Da using MS (Figure 2). By immunising SD rats with GnRH6-MBP recombinant protein and GnRH-OVA coupled protein, testicular atrophy was successfully induced by pathological section observation, and the mRNA expression levels of FSH receptor and LH receptor in the testes decreased. The serum levels of T, FSH, and LH showed a significant downward trend that lasted until 8 weeks after the first immunisation.

ELISA offers high sensitivity and specificity, rapid detection speed, minimal equipment requirements, and a broad spectrum of applications. Free from radioactive contamination, it facilitates ultra-micro and semi-micro quantitative analyses. It is currently the most widely used and rapidly developing enzyme immunology technology [30]. ELISAs are generally divided into direct, indirect, competitive, and blocking ELISAs. The indirect enzyme-linked immunosorbent assay (ELISA) is one of the most commonly used methods for antibody detection [31]. ELISA has high sensitivity and versatility; that is, it can be used to detect antibodies against various antigens in a species. Therefore, indirect ELISA was used to detect GnRH antibodies in rats.

In general, because the overall high or low OD value of the test result is prone to false positives or false negatives, the OD value is not suitable for direct judgment. Therefore, we used the OD450 nm values of the positive/negative samples as the criterion for the optimal detection conditions. Thus, an indirect ELISA method based on the GnRH6-MBP antibody was established. This method has high stability and sensitivity, providing conditions and guarantees for the subsequent immunisation of SD rats with the GnRH6-MBP protein and the construction of biomedical models.

Despite extensive research on GnRH vaccines, including their formulation, safety, immunogenicity, and long-term effects, this field is still in the early stages of development. This underscores the need for further exploration of the immune responses, lasting effects, and safety profiles of GnRH-based immunosterilisation to refine and enhance its application in animal sterilisation. In the future, we plan to conduct additional research on the side effects of GnRH immunisation and its impact on animal health and safety.

## 4. Materials and Methods

### 4.1. Animals and Materials

Eighteen healthy SD rats, aged 6 weeks, were purchased from Liaoning Changsheng Biotechnology Co., Ltd. (Shenying, China). *E. coli* BL21 competent cells were purchased from Beijing ComWin Biotech Co., Ltd. (Beijing, China). We used an in-house pMAL-c2x vector and goat anti-rat IgG antibody purchased from Abcam (Cambridge, UK). Dextrin Beads 6FF were purchased from Suzhou Boao Long Technology Co., Ltd. (Suzhou, China). A BCA Protein Assay Kit was purchased from Thermo Fisher Scientific (Waltham, MA, USA). Rat T, rat LH, and rat FSH enzyme-linked immunosorbent assay (ELISA) assay kits were purchased from Beijing Ruida Henghui Technology Development Co., Ltd. (Beijing, China). Tetramethylbenzidine (TMB) was purchased from Sigma-Aldrich (St. Louis, MO, USA).

### 4.2. Methods

#### 4.2.1. Construction of Recombinant Plasmid

**Figure 7:**
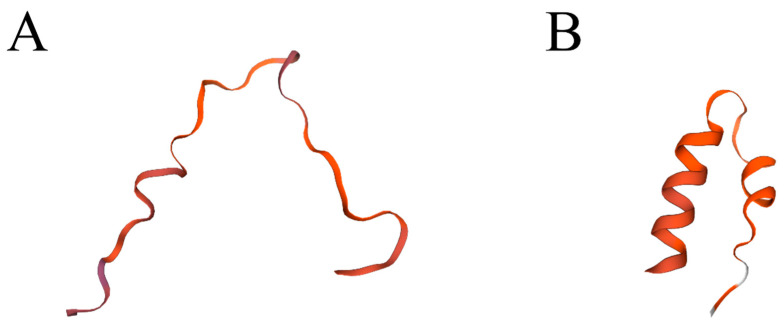
Swiss-model (**A**) GnRH6; (**B**) GnRH.

The construction and preparation of the recombinant plasmid the antigen of GnRH hexamer (GnRH6) and its preparation were designed based on the operational procedures described by Jung et al. [32], and a tandem repeat GnRH6 cDNA with a few amino acid substitutions was constructed (Figure 7A). The designed gene fragment was synthesised by Wuhan Ginko Biotechnology Co., Ltd. (Wuhan, China). First, the GnRH gene fragment in the pMAL-c2x plasmid was digested by separately using *EcoR*I and *SaI*I restriction enzymes, and the fragments were recovered for DNA ligation (Table 7). Then, the formed recombinant plasmid molecules were transformed into *E. coli* DH5α competent cells and grown in LB medium containing Amp (100 µg/L) at 37 °C for 16 h. The resulting recombinant plasmid is referred to as pMAL-c2x-GnRH6.

#### 4.2.2. Expression, Purification and Validation of the Target Protein

The pMAL-c2x-GnRH6 plasmid was transformed into BL21 competent cells. Single colonies were picked and cultured in LB medium containing ampicillin at 37 °C under shaking until the OD600 reached 0.6–0.8 The cells were induced with 1 mmol/L isopropyl β-d-1-thiogalactopyranoside (IPTG) for 6–8 h and harvested by ultrasonic disruption (150 W, 180 s). The supernatant and precipitate were collected and analysed for solubility using SDS-PAGE. Dextrin Beads 6FF were packed into a suitable chromatography column and equilibrated with five column volumes of the binding buffer. The sample was loaded onto equilibrated Dextrin Beads 6FF, and the flow-through was collected. Nonspecifically adsorbed proteins were removed by washing with 10–15 column volumes of wash buffer. The target protein, i.e., GnRH6-MBP, was eluted three times using 2 mL of elution buffer each time. The column was then equilibrated with three column volumes of binding buffer, followed by five column volumes of deionised water, and stored in 20% ethanol at equal volume and at 4 °C.

#### 4.2.3. Preparation of GnRH Synthetic Peptides

The GnRH antigen was prepared as described by Oonk et al. [33]. Natural mammalian GnRH consists of 10 amino acids with the peptide sequence Glu-His-Trp-Ser-Tyr-Gly-Leu-Arg-Pro-Gly-NH2 (Figure 6B) [34]. To facilitate conjugation of the GnRH molecule with the carrier protein, D-lysine was used to replace the 6th glycine of natural GnRH to form the conjugation site of GnRH and the carrier protein, using the -NH2 residue on D-lysine as the conjugation site. The protein sequence was thus Glu-His-Trp-Ser-Tyr-Lys-Leu-Arg-Pro-Gly-NH2. GnRH was chemically synthesised and combined with a dimer as the basic unit to prepare the GnRH antigens: pyroGlu-Glu-His-Trp-Ser-Tyr-Lys-Leu-Arg-Pro-Gly-Gln-His-Trp-Ser-Tyr-Gly-Leu-Arg-Pro-Gly-Cys-NH2. G6KT was chemically synthesised by Fulide Biotechnology Co., Ltd. (Wuhan, China).

The new protein configuration antigen, GnRH-OVA, was prepared by conjugating GnRH with ovalbumin as follows: (1) equal amounts of GnRH and OVA were dissolved in deionised water (15 mg/mL), and the mixture was stirred magnetically for 1 h; (2) 10 times the amount of carbodiimide (EDC) was dissolved in deionised water (0.2 g/mL); (3) after stirring for 1 h, solutions of GnRH and OVA were mixed, and the EDC solution was added dropwise to the mixed solution of GnRH and OVA using a dropper while magnetic stirring was maintained. The entire amount of EDC was added, and the mixture was continuously stirred at room temperature overnight; (4) the solution was dialysed using a 10,000 MWCO membrane in distilled water with water changes every 2 h for at least 8 h; (5) the sample was freeze-dried, and the conjugation efficiency was calculated based on the weight. The coupling efficiency was approximately 30%, i.e., yielding 0.5 mg GnRH/mg OVA.

#### 4.2.4. Establishment of an ELISA Method for GnRH6-MBP Antibody Detection

An initial indirect ELISA protocol was drafted, which was then refined through optimisation and the selection of specific conditions and steps.

Coating: Each well was filled with 100 μL of GnRH-MBP protein diluted in coating buffer and incubated at 4 °C for 12 h. Wells were washed four times with PBST, allowing the wash buffer to sit for 1 min each time, and tapped dry on absorbent paper after the final wash. Blocking: Each well received 120 μL of a 50 g/L non-fat milk solution, incubated at 4 °C for 10 h, followed by four washes with PBST, with each wash standing for 1 min before tapping dry on absorbent paper. Primary Antibody: Negative and positive sera were serially diluted with PBST, then 50 μL were added to the corresponding wells and incubated at 37 °C for 30 min. This was followed by four washes with PBST, with each wash standing for 1 min, and tapping dry on absorbent paper after the final wash. Secondary Antibody: 50 μL of diluted enzyme-conjugated antibody was added to each well and incubated at 37 °C for 30 min. The wells were then washed four times with PBST, allowing the wash buffer to sit for 1 min each time, and tapped dry on absorbent paper after the final wash. Development: 100 μL of TMB substrate solution was added to each well and incubated at 37 °C in the dark for 15 min. Termination: The reaction was stopped by adding 100 μL of stop solution to each well. Reading: The optical density (OD) at 450 nm was measured using an ELISA reader.

The chequerboard titration method was used to determine the optimal GnRH-MBP protein coating concentration and serum dilution ratio, with six antigen dilutions (0.5, 1, 1.5, 2, 2.5, 3 μg/mL). Positive and negative sera were diluted with PBST at ratios from 1:5 to 1:640, and 50 μL were added to the corresponding wells. Goat anti-rat IgG was diluted in PBST at four different ratios (1:5000, 1:10,000, 1:15,000, 1:20,000) and used for secondary antibody incubation. Three different blocking solutions were tested: 50 g/L bovine serum albumin, 50 mL/L fetal bovine serum, and 50 g/L non-fat milk solution. Five different antigen coating conditions were tested (4 °C for 8 h, 4 °C for 10 h, 4 °C for 12 h, 37 °C for 1 h, and 37 °C for 2 h). Four different development times were tested (5 min, 10 min, 15 min, 20 min). All conditions were explored, with the highest P/N ratio as the optimal condition.

Using the optimised indirect ELISA method, ten negative sera were tested to calculate the mean OD value at 450 nm (X̄) and standard deviation (SD). A sample was considered positive if its OD value was greater than X̄ + 3SD, negative if less than X̄ + 3SD, and equivocal if equal to X̄ + 3SD.

#### 4.2.5. ELISA Kit Sensitivity Test and Repeatability Test

Ten positive serum samples were diluted with PBST in 8 different ratios (1:640–1:81920) using the optimised indirect ELISA method. 50 μL was added to the corresponding wells. Samples were considered positive if P/N was greater than the cutoff for negative and positive controls.

To assess the reproducibility of the optimised indirect ELISA protocol, both intra-assay and inter-assay variability were evaluated. To ensure intraassay repeatability, single batch kits were prepared according to optimal conditions determined previously. Ten positive serum samples were tested in triplicate. The experimental procedure adhered strictly to the optimised steps of the indirect ELISA method. The standard deviation (SD) and coefficient of variation (CV) were computed from these results to quantify the variability within this single batch.

Simultaneously, inter-assay reproducibility was determined using three separate batches of test kits, also prepared under the same optimal conditions. Similar to the intra-assay evaluation, ten positive serum samples were tested with three replicates each. The indirect ELISA was performed according to the refined protocol, and the resulting data were used to calculate the SD and CV, providing a measure of variability across different batches. This dual approach ensures a comprehensive assessment of the assay’s reliability and precision.

#### 4.2.6. Experimental Design and Immunisation Procedure

Twenty-four healthy SD rats were randomly assigned to four groups: GnRH6-MBP treatment, GnRH-OVA treatment, a surgical castration group, and a black control group (six individuals per group). The GnRH6-MBP and GnRH-OVA compounds were mixed with Freund’s adjuvant at a 1:1 ratio and emulsified. Rats were immunised three times with 100 μg protein each time. Rats in the surgical castration group were surgically castrated, whereas those in the blank control group were injected with the same volume of saline. All rats had free access to food and water before and during the experiments.

#### 4.2.7. Collection of Samples

Blood samples were collected from rats before each immunisation for serum separation. Fourteen days after the third immunisation, rats were anaesthetised, blood was collected, and the testes and spleens were removed. The weight of the testes was measured in the blank control, GnRH6-MBP immunisation, and GnRH-OVA immunisation groups. This was performed to assess the impact of immunisation on testicular weight.

#### 4.2.8. Antibody Detection in Rat Serum

The GnRH6-MBP and GnRH-OVA were each diluted to 1 μg/mL using PBS buffer at pH 7.2, and 100 μL of the diluted solution was transferred to each well of an ELISA plate for incubation at 4 °C for 12 h. After discarding the coating solution, the plate was washed four times using phosphate-buffered saline with Tween detergent (PBST buffer; 0.05% Tween-20) at pH 7.4. Then, each well was blocked with 150 μL of 5% BSA blocking solution at 4 °C for 10 h and washed four times using PBST. Next, 50 μL of serum (diluted 1:500) was added to each well, followed by incubation at 37 °C for 30 min and washing four times using PBST. Subsequently, 50 μL goat anti-rat IgG (diluted 1:10,000) was added to each well for incubation at 37 °C for 30 min, followed by washing four times using PBST. Then, 50 μL of TMB substrate was added to each well, and the plate was incubated at 37 °C for 15 min in the dark. The reaction was stopped by adding 50 μL of stop solution to each well, and the OD450 nm was measured using an ELISA reader to calculate and analyse the experimental results.

#### 4.2.9. Paraffin Sections and Haematoxylin/Eosin Staining of Testis Tissues

The testes and spleens were washed using PBS and then fixed in 4% paraformaldehyde for 48 h after absorption on filter paper [35]. After fixation, the samples were washed with low-pressure running water to remove paraformaldehyde and dehydrated using a series of graded alcohol solutions. The samples were placed in xylene for approximately 20 min to remove residual alcohol and make them transparent. The testes and spleen were placed in moulds and first submerged in melted soft wax (52–54 °C) for 2 h, followed by hard wax I (56–58 °C) for 2 h and hard wax II (58–60 °C) for 2 h. Finally, the samples were embedded in moulds and allowed to cool and solidify using a biological tissue dehydrator.

The wax blocks were frozen on a freezing stage and sliced into 5-μm sections. The sections were floated on water at 40 °C and then picked up with anti-loose slides, followed by baking in a pathology tissue floating oven at 60 °C for 1 h. The slices were dewaxed in xylene for 1 h and then soaked in a series of alcohol solutions: 100% alcohol for 3 min, 95% for 3 min, and 75% for 3 min, followed by washing under running water for 10 min. The hydrated sections were dried and stained with haematoxylin for 3 min, followed by washing under running water for 3 min. The sections were then stained with eosin for 2 min, followed by washing under running water for 3 min to terminate the staining. Finally, the sections were dehydrated in a series of alcohol solutions: 75% alcohol for 2 min, 95% for 2 min, and 100% for 2 min, after which they were made transparent using xylene and mounted with neutral resin for observation and photography using a microscope.

#### 4.2.10. Measurement of Serum T, LH, and FSH

Following the manufacturer’s instructions, test samples and experimental reagents were added sequentially. After the reaction was complete, the optical density (OD) of each well was measured at OD 450 nm using an ELISA reader, and statistical analysis was performed.

#### 4.2.11. Statistical Analysis

Graphpad Prism 8.0.2 software was used to analyse differences between groups, with a *t*-test to compare differences between the two groups and one-way analyses of variance to compare differences between multiple groups. *p* < 0.05 indicates statistical significance.

## 5. Conclusions

In this study, the recombinant plasmid pMAL-c2x-GnRH was successfully prepared by electrophoresis in agarose gel. At the same time, the synthetic GnRH peptide with a molecular weight of 2467.72 was successfully obtained, and the purity was higher than 98% by HPLC.

Using GnRH6-MBP recombinant protein as coating antigen, the indirect ELISA method based on GnRH-MBP recombinant protein antibody detection was preliminarily established. It was proven that the method had good repeatability, high sensitivity, and could detect GnRH antibody in SD rat serum.

GnRH6-MBP recombinant protein was used to evaluate the animal immune effect in SD rats, and the results showed that GnRH6-MBP recombinant protein had a good immune castration effect.

## Figures and Tables

**Table 1:** Determination of optimal coating antigen mass concentration and serum dilution.

Serum Dilution Folds	Samples	Coating Antigen Mass Concentration (μg/mL)					
		0.5	1	1.5	2	2.5	3
1: 5	P	0.836	1.159	1.191	1.310	1.258	1.287
	N	0.124	0.131	0.125	0.132	0.132	0.121
	P/N	6.742	8.847	9.528	9.924	9.53	10.636
1: 10	P	0.635	0.852	0.895	1.012	0.985	0.997
	N	0.111	0.121	0.125	0.115	0.131	0.131
	P/N	5.721	7.041	7.16	**10.896**	9.908	10.092
1: 20	P	0.520	0.629	0.732	0.921	0.901	0.923
	N	0.079	0.091	0.102	0.091	0.098	0.096
	P/N	6.582	6.912	7.718	10.121	9.194	9.615
1: 40	P	0.481	0.588	0.679	0.836	0.849	0.899
	N	0.074	0.077	0.098	0.080	0.099	0.091
	P/N	6.500	7.636	6.929	10.450	8.576	9.879
1: 80	P	0.416	0.549	0.689	0.780	0.811	0.803
	N	0.073	0.069	0.085	0.078	0.084	0.088
	P/N	5.699	7.957	8.106	10.000	9.655	9.125
1: 160	P	0.430	0.506	0.596	0.741	0.765	0.772
	N	0.062	0.065	0.078	0.073	0.073	0.079
	P/N	0.935	7.785	7.641	10.151	10.479	9.772
1: 320	P	0.386	0.552	0.576	0.703	0.697	0.719
	N	0.055	0.060	0.065	0.068	0.063	0.071
	P/N	7.018	9.200	8.862	10.338	11.063	10.127
1: 640	P	0.379	0.486	0.555	0.663	0.645	0.656
	N	0.053	0.050	0.058	0.065	0.062	0.065
	P/N	7.151	9.720	9.569	10.200	10.403	10.092

**Table 2:** Determination of optimal dilution of HRP-conjugated goat anti-ferret antibody.

Samples	Dilution			
	1:5000	1:10,000	1:15,000	1:20,000
P	1.856	1.574	1.098	0.854
N	0.156	0.128	0.117	0.098
P/N	11.897	**12.297**	9.385	8.714

**Table 3:** Determination of the optimal blocking solution.

Samples	Blocking Solution		
	50 g/L Bovine Serum Albumin	50 mL/L Bovine Serum Albumin	50 g/L Non-Fat Milk Solution
P	1.472	1.528	1.513
N	0.123	0.120	0.118
P/N	11.967	12.733	**12.822**

**Table 4:** Determination of the optimal coating conditions for the antigen.

Samples	Coating Conditions				
	4 °C 8 h	4 °C 10 h	4 °C 12 h	37 °C 1 h	37 °C 2 h
P	1.359	1.452	1.568	1.421	1.499
N	0.111	0.117	0.120	0.108	0.123
P/N	12.243	12.410	**13.067**	12.981	12.187

**Table 5:** Determination of the optimal TMB colour time.

Samples	Colour Time/min			
	5 min	10 min	15 min	20 min
P	1.633	1.745	1.895	1.942
N	0.102	0.109	0.123	0.121
P/N	16.010	16.009	15.407	**16.050**

**Table 6:** Repeatability assay.

Samples	Intra-Assay Variability			Inter-Assay Variability		
	Mean	SD	CV/%	Mean	SD	CV/%
A	1.572	0.052	3.296	1.588	0.011	0.694
B	1.592	0.064	4.026	1.621	0.043	2.627
C	1.593	0.067	4.212	1.578	0.043	2.744
D	1.591	0.036	2.232	1.683	0.033	1.945
E	1.674	0.033	1.949	1.692	0.057	3.339
F	1.650	0.027	1.614	1.657	0.074	4.477
G	1.632	0.059	3.633	1.666	0.089	5.358
H	1.649	0.033	1.971	1.549	0.044	2.860
I	1.579	0.045	2.818	1.555	0.035	2.250
J	1.632	0.043	2.636	1.539	0.031	2.023

**Table 7:** Amino acid sequence and restriction site of GnRH6.

DNA Sequence of GnRH Hexamer												
5′	GAATTC	CAA	CAT	TGG	AGT	GGT	GGC	TTA	CGT	CCT	GGT	
	*EcoR*I	Gln	His	Trp	Ser	Gly	Gly	Leu	Arg	Pro	Gly	
	GGC	AGT	AGC	GAA	CAC	TGG	AGC	TAC	GGT	TTG	AGA	
	Gly	Ser	Ser	Glu	His	Trp	Ser	Tyr	Gly	Leu	Arg	
	CCC	GGG	AGC	GGG	CAG	CAC	TGG	AGC	TAT	GGG	CTG	
	Pro	Gly	Ser	Gly	Gln	His	Trp	Ser	Tyr	Gly	Leu	
	AGG	CCA	GGA	GGT	GGA	AGT	GAG	GAT	AGT	TAT	GGA	
	Arg	Pro	Gly	Gly	Gly	Ser	Glu	Asp	Ser	Tyr	Gly	
	CTA	CGG	CCG	GGC	AGT	CAG	CAC	TGG	AGC	TAC	GGC	
	Leu	Arg	Pro	Gly	Ser	Gln	His	Trp	Ser	Tyr	Gly	
	CTG	CAG	CAT	TGG	AGT	TAT	GGA	TTA	CGG	CCA	GTCGAC	3′
	Leu	Gln	His	Trp	Ser	Tyr	Gly	Leu	Arg	Pro	*Sal*I	

## Data Availability

Original data used and generated in this study are available from the corresponding authors on request with a completed Data Transfer Agreement. Informed consent was obtained from all subjects involved in the study. Written informed consent has been obtained from the patient(s) to publish this paper.
